# Validating self-reported Toxic Release Inventory data using Benford's Law: investigating toxic chemical release hazards in floodplains

**DOI:** 10.3389/fpubh.2024.1342510

**Published:** 2024-06-17

**Authors:** Kristin Osiecki, Syed Hussaini, Apostolis Sambanis, Logan Quinsey, Chloe Liew

**Affiliations:** ^1^Center for Learning Innovation, University of Minnesota Rochester, Rochester, MN, United States; ^2^Emergency Management and Resilience Planning Graduate Certificate Program, School of Public Health, University of Illinois at Chicago, Chicago, IL, United States

**Keywords:** Benford's Law, Toxic Release Inventory, flood risk, vulnerable populations, environmental justice

## Abstract

**Introduction:**

Acute and long-term health impacts from flooding related toxic chemical releases are a significant local health concern and can disproportionately impact communities with vulnerable populations; reliable release data are needed to quantify this hazard.

**Methods:**

In this paper, we analyze US Federal Emergency Management Agency designated floodplain data and US Environmental Protection Agency Toxic Release Inventory (TRI) data to determine if geographically manipulated databases adhere to Benford's Law.

**Results:**

We investigated multiple variants and discovered pollution releases adhere to Benford's Law and tests which thereby validates the self-reported toxic release dataset.

**Discussion:**

We find that Benford's Law applies to self-reported toxic chemical release and disposal data, indicating a lack of widespread data errors or manipulation.

## Introduction

The United States Environmental Protection Agency (USEPA) relies on a volunteer reporting of pollution emissions by manufacturing facilities utilizing chemicals listed on their Toxic Release Inventory (TRI). This honor system is often questioned for its' accuracy in reporting such emissions to ensure regulatory compliance to minimize hazardous releases and resulting human exposure. To determine the effectiveness of this reporting method, environmental health scientists are investigating the potential Benford's Law (BLs) to discover anomalies in TRI reported levels of pollutant. For example, in 2006 Marchi and Hamilton (1), compared self-reporting air constituents in relation to ambient air monitoring collected by the USEPA. Testing BL using air pollution samples, they determined that BLs is potential tool to discover discrepancies with under-reporting (1). Utilizing BLs to investigate pollution discharges show increasing promise (1–4). This study examines the potential of using BLs with TRI emissions in designated flood zones in the United States to determine the efficacy of discovering abnormalities associated with under-reporting to identify exposure concerns in regard to flood related disaster.

BLs looks at the frequency of the first digits in real life databases and that the distribution of such digits that range from 1 to 9 in big data sources are not randomly distributed but follow distinct probability curve with the lower digits occurring more often than the higher digits (5). The first digit law was first discovered by Simon Newcomb in 1881, an astronomer looking at the distribution of numbers in logarithm tables and then rediscovered by its namesake, Frank Bedford, a physicist in 1938 (5). In 2000 it was show that BL could be used to detect database anomalies, including data errors or manipulation (3) and was later used to this effect in financial databases to discover credit card fraud (6). Its uses have expanded as a potential investigative tool in environmental health datasets. For example, in the emergency management field. Using its ability to analyze large BLs has been applied to the prediction of natural hazards, including cyclones and hurricanes; in particular, researchers analyzed the historical records of cyclone occurrences using BLs to seek possible explanations for changes in weather patterns, which could potentially give insight into the impacts of climate change (7).

In this study, we examine the application of BLs on the USEPA TRI data source looking at the use and disposal of toxic chemicals by manufacturing facilities located in floodplains. The concern over these floodplains is especially relevant due to the increase in severe weather from climate change and a higher risk of flooding impacting manufacturing sites, which can lead to toxic chemicals leaching off site and impacting surrounding communities. Since its creation by the Emergency Planning and Community Right-to-Know Act (EPCRA), the TRI has collected data on reported toxic chemical releases and pollution prevention activities by industrial facilities at the state and federal levels (8). As of 2019, there are 770 listed chemicals within 33 chemical categories which are chosen based on their carcinogenic, acute human health effects, or adverse environmental effects (9). The list of TRI chemicals is not all encompassing with mostly large manufacturers, metal mining, electric power, chemical plants, and hazardous waste treatment

This is a provisional file, not the final typeset article facilities: and with such a large amount of data on toxic releases, the TRI can help make informed decisions within low-income and minority communities regarding emergency management for preparation, response, and mitigation of these chemical spills (10). This risk is not theoretical; a recent example of a flooding event in the form of a storm surge inundated TRI sites in the Houston Metropolitan Area during Hurricane Harvey in August of 2017. The total economic cost of the storm was estimated to be between $81 to $108 billion, and tens of thousands of homes, along with over 700 businesses, were damaged by this extreme weather event throughout southeast Texas (11). After the massive amounts of flooding from Harvey's torrential rains and storm surge, hundreds of industrial facilities released excessive amounts of toxic chemicals into surrounding waterways and neighborhoods. After the release of dozens of tons of industrial toxins– including benzene, vinyl chloride, and other human carcinogens –the long-term human health consequences from flood-induced TRI site releases continue to be a major concern (11).

The potential for mitigating future risk of toxic chemical releases into the environment has turned pattern analysis of TRI designated facilities in floodplains into an emerging field of research. Given the potential long-term health impacts of toxic chemical use and disposal exposure among nearby residential communities, it is valuable to understand if BLs can help identify anomalous chemical release data from facilities within these high-risk areas. With the increase of extreme flooding, tropical storms, and hurricanes due to climate change, it is essential to properly allocate billions of dollars of resources for mitigation and emergency response planning to minimize damages and negative public health impacts. The evaluation of potential chemical releases resulting in hazardous exposures is a valid concern during emergency response and mitigation. Another area to consider when discussing climate change and the increasing occurrence of extreme weather events are how exposures to toxic chemicals and disparities in emergency preparedness and response measures disproportionately affect vulnerable populations, e.g., communities of color, low-income neighborhoods, immigrant groups, and indigenous people. In emergency management, populations that are at greater risk of negative impacts from a natural disaster are considered vulnerable as quantified by an index that consists of numerous U.S. Census indicators such as age, housing, non-English speaking residences, etc. (12). Institutional level constructs further exasperate this inequity due to structural racism, lack of neighborhood infrastructure, health disparities, lack of social and political capital, and fewer evacuation resources (13).

Numerous studies show that racial/ethnic minority communities suffer from greater environmental burdens because they live in areas with elevated environmental hazard exposure levels (2, 14–22). A recent study used spatial distribution mapping technology to demonstrate that environmental hazards increase in concertation the closer they are to urban centers and pollution sources (2). Furthermore, such proximity to sources of environmental exposure is linked with negative health outcomes with a mutagenic risk of about 18 times greater than recommended (23). Low-income minority communities are located near high-polluting industries, hazardous waste facilities, and incinerators (24, 25), and regional investigations confirm a relationship between TRI locations and communities of color (25, 26). TRI sites in flood plains that reside in areas with vulnerable populations increase the potential of negative human health outcomes, unexpected acute chemical emergencies, and longer periods of recovery.

The USEPA relaunched the climate change indicator website, after a 4-year hiatus, which provides general information, data, and mapping tools to better understand extreme climate changes affect public health and the physical environment (27). Excessive heat waves, droughts, wildfires, flooding, hurricanes, and rising ocean levels are no longer predictions but evidence-based realities with an accelerating increase in the number and severity of natural disasters. Associated with the increase of extreme events, is the increasing toll in both damage costs and human lives. Between 1980 and 2020 the United States experienced 290 natural disasters that exceeded $1 billion, which were cumulatively responsible for almost 14,500 deaths with total costs of $1.9 trillion (28). From 1980 to 1989 the U.S. averaged 2.9 billion-dollar events annually. By the 2016 to 2020 period the number of events more than quintupled, with an average of 16.2 events annually. 2020 set a new record with 22 natural disasters that exceeded $1billion in damages (28). This trend demonstrates the increasing need to be able to predict in which spaces climate impacts will have the most adverse health consequences.

To help mitigate the changing climate, the U.S. Federal Emergency Management Agency (FEMA) developed a variety of prediction models for emergency preparedness, response, mitigation, and recovery. For example, FEMA's Hazus Program utilizes geographic information system software to estimate risks and costs from earthquakes, floods, tsunamis, and hurricanes utilizing historical data. Regions and neighborhoods with vulnerable populations can be identified to increase community resilience to these events (29). Likewise, the FEMA Interagency Modeling and Atmospheric Assessment Center models atmospheric dispersion in relation to acute chemical emergencies (30). Models are indispensable tools to help understand past events and, more importantly, predict natural disaster outcomes as the number and severity of climate change induced weather events continue to grow.

**Figure 1:**
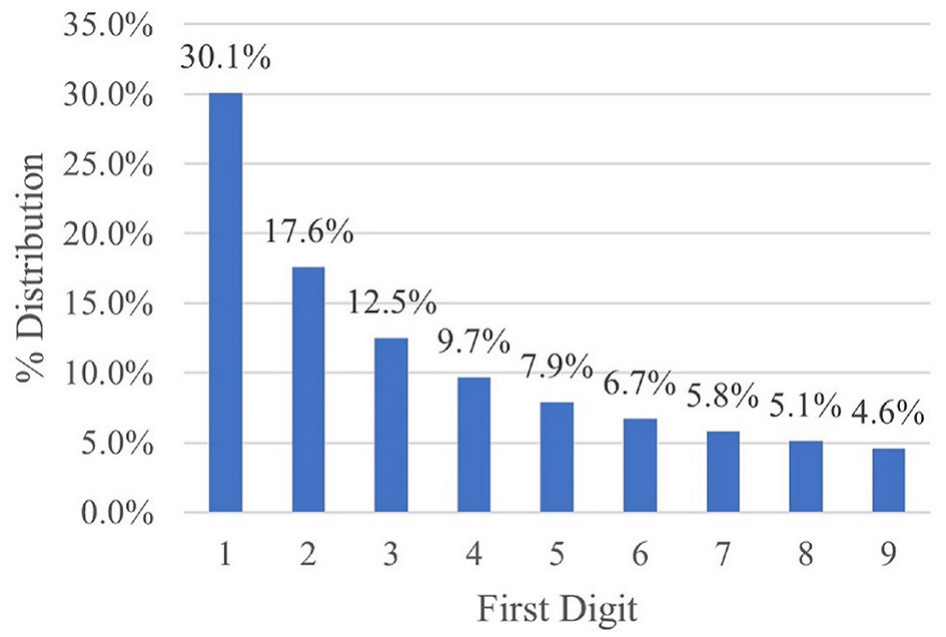
Benford's Law: percentage of time digits 1 through 9 are expected to occur in the first position.

Investigating BLs in relation to flooding events and TRI locations has the possibility to be a prediction tool for emergency preparedness and response. BLs characterizes the distribution of first digits, second digits and the first two digits within large datasets. Simon Newcomb first described the tendency for numbers with the first digit of 1 to be observed more often than other numbers (i.e., first digits of 2, 3, and so on), and later re-discovered by Frank Benford (31). BLs allows for the prediction of the leading digit(s) distribution in certain datasets, and it is more accurately applied when there are multiple magnitudes that the data covers evenly (7). After the initial discovery of BLs in 1938, subsequent research has been focused on potential applications for its use. The logarithmic distribution described by BLs has been found to follow large numerical data from a variety of natural and social phenomena, ranging from extreme weather events to fraud detection (32). BLs natural disaster studies find that BLs can be used to examine data quality and homogeneity to ensure variables chosen for analysis are the best for decision making (7). BLs relies on the log10 distribution within the data with a right-tail skewed distribution of 1 – 9 (with no unit value), which is commonly found with environmental health data sets such as air and water samples. BLs adhere to a percent scale in the form of a histogram (Figure 1) from 1 – 9 on the x-axis and the percent on the y-axis (5).

Although BLs are used to analyze a myriad of larger datasets to unearth anomalies, it remains a highly contested tool due to the elusiveness in proving the law within mathematical theory or statistical methods. Benford's Law Strikes Back by Berger and Hill (33) provided the derivation to explain the BLs phenomenon. Over the past 15 years, BLs continues to be further scrutinized to understand the logic behind the distribution frequency (33–35) examining goodness of fit, severity evaluations, and arithmetic/geometric means. These contributions provide greater insight into BLs, however, a complete explanation of the workings of the law is still under investigation.

Our study expands upon previous BLs natural disaster research looking at cyclones, hurricanes, earthquakes, and TRI site emissions. We analyze FEMA designated floodplain data in relation to TRI locations to determine if BLs adheres to geographically manipulated databases looking at space and place. Finding abnormalities within the TRI database can help predict potential emergency situations within floodplains associated with the increase of extreme weather events.

## Methods

This research's methodology focuses on examining TRIs in floodplains that are identified after an inundation event. The analysis was done by compiling and analyzing various federal governmental data sets. Three primary data sets were analyzed: TRI site locations; TRI site release and disposal values; and historical federally declared flooding disasters. These data were cross referenced by zip code and by county and visualized in ArcMap to highlight the potential risk posed to an area from both flooding and a resultant acute chemical emergency.

A list of inundation likelihood from FEMA was compiled by comparing the number of TRI facilities in the USEPA dataset (10) present in a particular area with the number of federally declared flood disasters in that region. The locations of the Toxic Release Inventory sites, which include a specific street number, were assigned to floodplains delineated with ESRI ArcMap geocoding by aggregating the point data into state and county boundaries. This allowed for exact mapping of the site locations. The database was then exported into IBM Statistical Product and Service Solutions (SPSS) software to perform descriptive statistical analysis. A total of 4,145 TRI sites in the United States were found to be in floodplains.

Microsoft Excel 2010 with Benford's Law and test formulas were used to analyze several non-uniform variables associated with these sites to determine if the information adhered to BLs first digit order and multiple variants (36). To validate our data analysis for TRI sites in floodplains, and to ensure inherent bias was removed based on a possible increased number of flood plains in urban versus rural areas, all TRIs in Alabama and California were combined into a dataset totaling 7,844 sites and subjected to BL analysis. TRI facilities, total on- and off-site disposal or other releases in U.S. pounds by zip code were gathered within a range of 1 - 885,683,717 U.S. pounds (10). Types of disposals and releases include but are not limited to air emissions, surface water discharges, underground injections, and landfill disposal. 7,684 TRI Sites with at least 1-pound total onsite releases or other disposal locations. Using Microsoft Excel 2010, duplicate zip codes were removed leaving a total of 7,393 zip codes that lie within declared flooding disasters by county. Total pounds of chemical releases for these 7,684 sites were analyzed to see if the release values conform to BLs. According to Nigrini (37), there are multiple variants to examine BLs of conformity including the first digit, the second-digit, or the first two digits. The first Equation (1) examines the first digit (38). Then, chemical releases were analyzed by BL2 in which the second digit is also examined with the Equation (2). Lastly, the releases were investigated using the BL12 Equation (3) that looks at the first and second digit. The formulas for the digits include D1 representing the first digit, D2 the second digit, and D1D2 the first-two digits of a number and Prob is the probability of observing the event in parentheses (37).


(1) $$\begin{array}{l} \begin{array}{c} Prob\left(D_{1}=d_{1}\right)=\log\left(1+\frac{1}{d_{1}}\right);d_{1}\in \left\{1,2,....,9\right\} \end{array} \end{array}$$

(2) $$\begin{array}{l} Prob\left(D_{2}=d_{2}\right)=\overset{9}{\underset{d_{1}=1}{\sum }}\log\left(1+\frac{1}{d_{1}d_{2}}\right);d_{2}\in \left\{0,1,.......9\right\} \end{array}$$

(3) $$\begin{array}{l} Prob\left(D_{1}D_{2}=d_{1}d_{2}\right)=\log\left(1+\frac{1}{d_{1}d_{2}}\right);d_{1}d_{2}\in \left\{10,11,.....99\right\} \end{array}$$

Each Benford's Law test for BL1, BL2, and BL12 were investigated for significance using the Z-statistic for outlier detection (Equation 4), with Z = the standard statistic, EP = the expected proportion, AP = the actual proportion, and *N* = the number of records. The (1/2N) is a continuity correction term and is used if it is smaller than the first term in the numerator. Nigrini (37) states that Z-statistic tests if the actual proportion for a specific digit is different than the digits expected with BLs. This test is used to test the null hypothesis of conformity.


(4) $$\begin{array}{l} z=\frac{|AP-EP|-\left(\frac{1}{2N}\right)}{\sqrt{\frac{EP\left(1-EP\right)}{N}}} \end{array}$$

Druică et al. (39) believe that the Mean Absolute Deviation (MAD) (Equation 5) which examines conformity where *Obs*_*k*_ = observed frequency, *Exp*_*k*_ = expected frequency of the class k, and *N* is the sample size is a better test than looking at significance when looking at the null hypothesis (36, 39).


(5) $$\begin{array}{l} MAD=\frac{1}{90}\overset{99}{\underset{k=10}{\sum }}\frac{\left|Obs_{k}-Exp_{k}\right|}{N} \end{array}$$

Additionally, FEMA Disaster Declaration summaries were downloaded from 1953 to 2021 (current), which totaled 61,898 total natural disasters (40). Using Microsoft Excel 2010, disasters prior to 1990 and those that did not involve flooding events were removed, leaving a total of 28,078 flooding disaster declarations. Duplicate zip codes were eliminated, leaving a total of 3242 U.S. counties with TRI facilities. These data were used to create visualizations in GIS ArcMap of counties with flooding disaster declarations overlaid with zip codes containing at least one TRI site with at least 1 pound total onsite or other disposal location releases that lie within floodplains.

## Results

**Figure 2:**
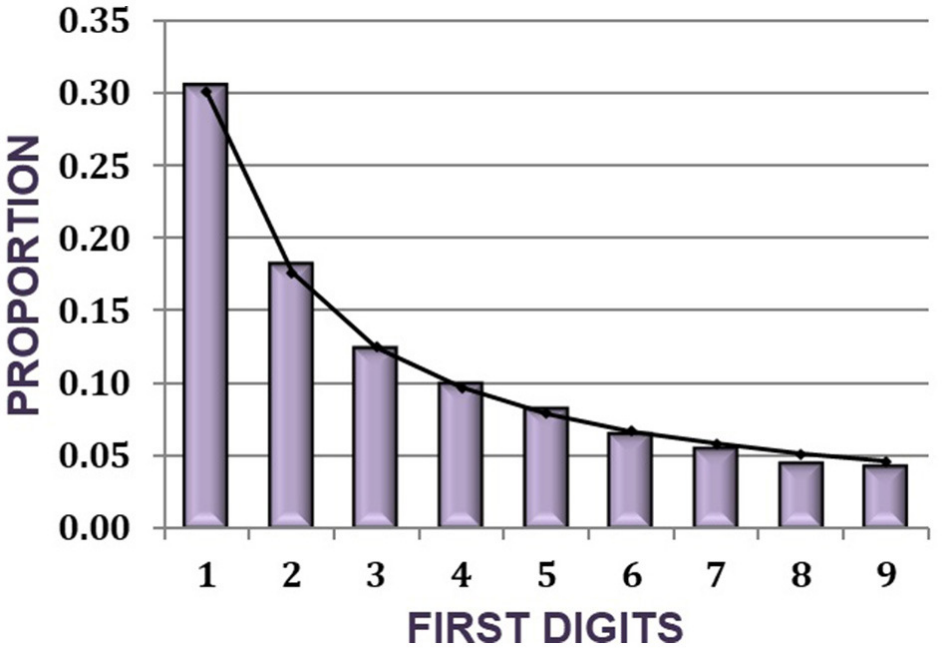
BL breakdown of chemical release: first digit distributions of chemical release amounts for 7,684 TRI facilities located in declared flooding disasters.

The USEPA TRI database provides numerous options to download data by facility, chemical, industry type, and geography. Data was collected by total pounds of chemical releases onsite and other offsite disposal by zip code. A total of 7,684 TRI sites across the U.S. for total onsite and offsite chemical releases fit the first digit law for digits 1 – 9. The Figure 2 shows the skewed nature of the chemical release data with the percentages closely aligned with the BLs percentages.

To determine goodness of fit of observed data vs. expected data, the following hypothesis was developed:

H_0_ (null hypothesis)**:** The TRI facilities pollution emissions distribution conforms to Benford's Law.

H_1_ (alternate hypothesis): The TRI facilities pollution emissions distribution is different from Benford's Law. The TRI facilities pollution emissions conform to Benford's Law.

One way to test the null hypothesis of conformity, a chi-square test looks at expected vs. observed outcomes. However, this test has an excessive power issue when working with larger data sets of more than 5,000 records (36). This study involves more than 7000 records; therefore, the Z-statistic and MAD are utilized to determine conformity instead of the chi-square test. In Table 1, the results of the BL first digit for 1,…,9 are shown with the corresponding Z-statistic, and MAD.

**Table 1:** Benford's Law for the first digit outcomes for the pollution generated by 7,684 TRI sites.

**First digit**						
**First**	**Count**	**Actual**	**Benford**	**Difference**	**AbsDiff**	**Z-stat**
1	2347	0.305	0.301	0.004	0.004	0.838
2	1397	0.182	0.176	0.006	0.006	1.306
3	953	0.124	0.125	−0.001	0.001	0.221
4	765	0.100	0.097	0.003	0.003	0.769
5	629	0.082	0.079	0.003	0.003	0.852
6	503	0.065	0.067	−0.001	0.001	0.496
7	419	0.055	0.058	−0.003	0.003	1.271
8	342	0.045	0.051	−0.007	0.007	2.614
9	328	0.043	0.046	−0.003	0.003	1.260
				**MAD** **=**	**0.00345**	

The bold indicates that the mean absolute deviation is significant.

The Z-statistic looks at a two-sided *p*-value for each observed proportion and the BLs proportion (41). It is important to note that in this instance, the *p*-value is not a measure of significance but an absolute value to use for comparison to accept or reject the null hypothesis. Since our z-statistics are >2.77 and <0.0056 (Table 1), we will not reject the null hypothesis because both proportionate values equate to each other (41).

The MAD test is not reliant on the size of a dataset and commonly used for big data. The results are absolute and are measure by a standard of conformity that was created by Nigrini and his interpretation of BLs based on his experience working with datasets (42). The MAD critical score for the first digit analysis equals 0.00345. This is within the range of 0.000 to 0.0006 which means that it is close to conformity.

We then investigated BL2 with results presented in Table 2. Even though the statistic is somewhat higher for some of the buckets, the interpretation of the Z-statistic and the MAD is the same for BL2.

**Table 2:** Benford's Law for the second digit outcome for the pollution generated by 7,684 TRI sites.

**Second digit**						
**Second**	**Count**	**Actual**	**Benford**	**Difference**	**AbsDiff**	**Z-stat**
0	1111	0.145	0.120	0.025	0.025	6.713
1	817	0.106	0.114	−0.008	0.008	2.066
2	822	0.107	0.109	−0.002	0.002	0.497
3	744	0.097	0.104	−0.007	0.007	2.130
4	699	0.091	0.100	−0.009	0.009	2.703
5	783	0.102	0.097	0.005	0.005	1.533
6	675	0.088	0.093	−0.006	0.006	1.641
7	715	0.093	0.090	0.003	0.003	0.809
8	661	0.086	0.088	−0.002	0.002	0.456
9	656	0.085	0.085	0.000	0.000	0.100
				**MAD** **=**	**0.00665**	

The bold indicates that the mean absolute deviation is significant.

For BL12, the highest z-statistic for 10,11,…,98,99 (table not included) is 3.06 with the remaining z-scores below 2.77. The MAD critical score for the first- and second-digit analysis of the pollution data equals 0.00101. This is within the range of 0.000 to 0.012 for the first two digits which means that it is close to conformity (42).

**Figure 3:**
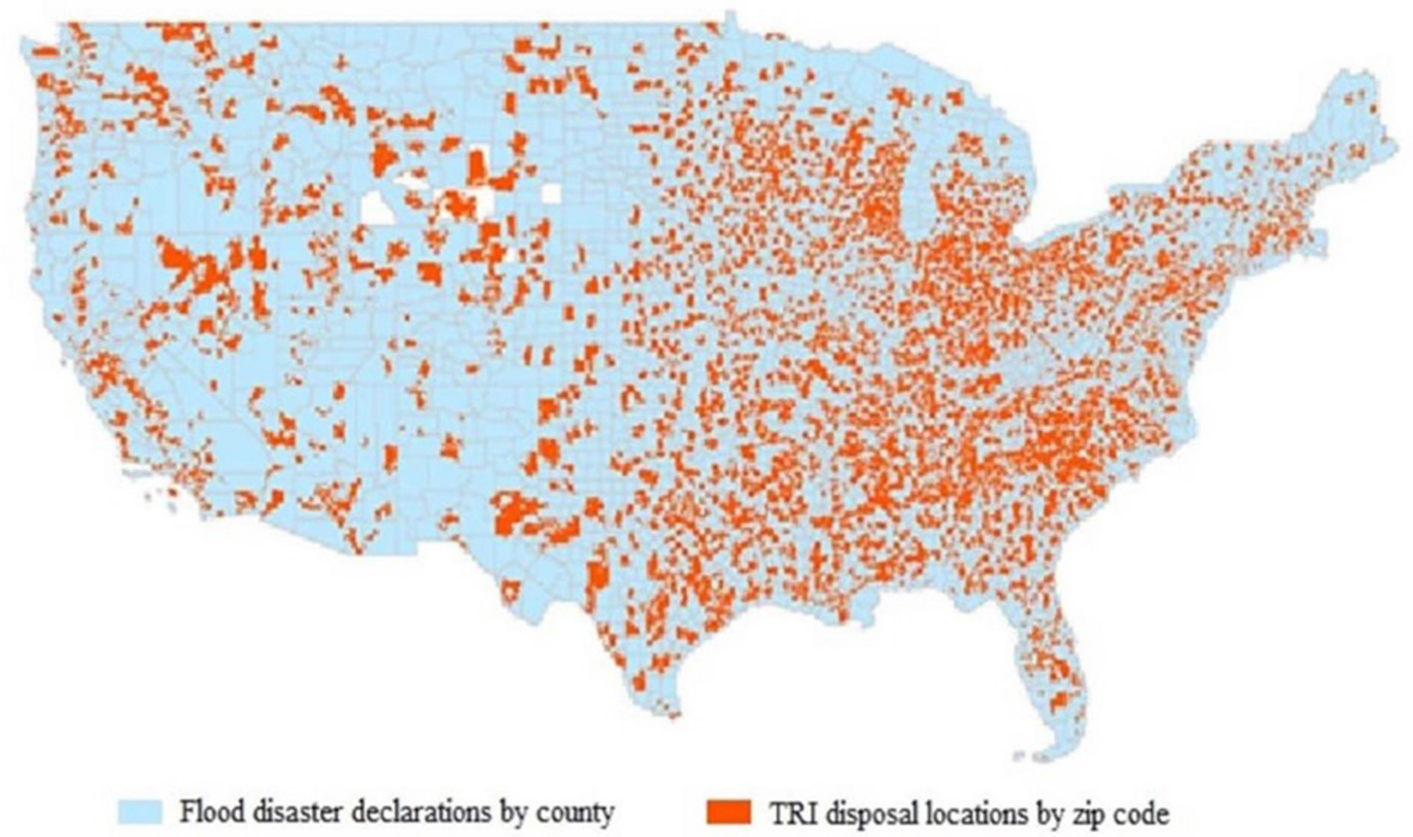
TRI disposal locations and historical disaster flooding declarations: TRI onsite or offsite disposal locations, 7,393 zip codes with no duplicates for map; 3,242 county flooding disaster declarations, 1990 to 2021 with no duplicates for map.

As shown in the ArcMap visualization (Figure 3), the majority TRI disposal locations lie in counties that had a disaster flood declaration between 1990 and 2021.

## Discussion

Health disparities research has focused primarily on racial and socioeconomic factors in relation to an increase of negative health outcomes. Although neighborhood characteristics and the concept of built environment have been shown to affect individual health, measuring the effects of environmental risks on health has been a less developed area of disparities research (43). Emergency management reliance on social vulnerability is complex and varies between scales (e.g., County, census block), however; the national scale of this study highlights the widespread of TRI manufacturing facilities in flood prone areas. This suggests that climate change indicators might be an inclusive factor when examining vulnerability, especially when socioeconomic status and race are recognized to be the two largest contributing factors to social vulnerability (44). The impacts of race and socioeconomic status on social vulnerability were particularly evident in the response to Hurricane Katrina Toxic chemical exposure was a major concern given the industrial base of the New Orleans area (45) and this area has yet to fully recover. Recognizing methodologies that can combine environmental health risk factors and social vulnerability provides necessary details during extreme climate change events. However, this is more challenging due to the multidisciplinary approaches to these models. Almost every county throughout the U.S. has experienced at least one flooding disaster declaration over the past 30 years and contains zip codes with TRI sites. A total 4,145, or ~78% of total TRI facilities are in designated floodplains. Since 1980, all fifty states have been impacted by at least one natural disaster that exceeded $1 billion which continues to increase with the intensity and number of disasters. USEPA policy suggests working with social scientists in environmental justice research to better understand the complex social structures within a community (46), and environmental scientists/emergency managers that investigate climate change, extreme weather events, and pollution modeling.

In regards to BLs and first digit outcomes associated naturally occurring events, there is a possible shift from predictability to detection (7). The implications of BLs are significant to the study of health and place because the data for both onsite and offsite TRI hazardous chemical releases and disposals was not uniform, therefore, independent, and conformed to BLs irrespective of the units of data as well as their source. This implies that BLs is a universal property of real-world measurements for TRI releases and disposals. It is important to note that although the possibility of utilizing BLs to determine anomalies within voluntary TRI pollution discharge reporting, the mechanisms behind BLs are still not fully understood. The chi-square goodness-of-fit-test confirmed conformity of the TRI pollution emissions data with BLs but utilizing this test is also highly criticized that conformity is achieved by a problem of excessive power inherently apparent in large datasets (35). Discrepancies in these datasets might falsely detect anomalies that do not exist. Emerging research that explores different goodness of fit approaches such as severity testing prove to be promising (35). Therefore, we investigated the z-statistic and the MAD critical score for BL1 and found the result supported the goodness of fit for the first digit. However, because of known issues with BL1, we expanded our analysis to examine BL12 and BL2, and discovered that the pollution discharge data from 7,648 TRI sites met the criteria to not reject the null hypothesis for the z-statistic and the MAD critical score showed close conformity. These outcomes provide further evidence that BLs can discover anomalies with self-reporting pollution discharges, and with summation, the ability to find over-reported or abnormal values to find TRI sites that might have mis-reported their numbers.

The number of TRI sites vulnerable to flooding in this study should be considered a conservative count; limitations of flood mapping throughout the U.S. is a known issue (47), and we can expect these numbers to rise when the flood zones for TRI locations are updated (48). Future research should investigate the exact flooding emergency planning protocols that TRI facilities practice state-to-state.

Chemical facilities operating at safer flooding standards than their state counterparts should have their standards reviewed to determine the applicability from facility to facility. This would lend a hand in determining which specific protocols should be revised and improved to contribute to a safer chemical facility regardless of specific region. Besides TRI facilities, health care facilities locations have also been documented in flood plains. Interactive floodplains maps could be used to reliably identify healthcare facilities vulnerable to chemical release from flood hazards. TRI facilities that may result in an acute chemical release during a flood, first responders tasked in controlling the emergency and assisting human exposures, and healthcare facilities responsible for quick and immediate care, would benefit from a shift to reliable data sources for predicting acute, events in flood areas that include chemical releases (49).

To further improve this study, the incorporation of the disaster loss data for analysis, in conjunction with socioeconomic data, is a critical performance assessment technique that can effectively determine current approaches and compare the accuracy of other methods for identifying high-risk areas (43). Further studies should explore the health risks posed by toxic chemical manufacturing hazards exposed to natural flood hazards to places with the most vulnerable populations who have the least amount of capacity to prepare, respond and recover (e.g., non-white and lower socioeconomic groups).

## Conclusion

The values for TRI Sites' total onsite release or other offsite disposal locations of toxic chemicals with at least one pound recorded align with BL, BL2, and BL12 and could indicate there are no database anomalies. This includes data errors or manipulation with a required federal program that is reliant on the honor system of these facilities to report their releases. The implication is that TRI release data may be generally reliable for conducting local risk assessments, although this study does not confirm the reliability of any individual release values. Almost all the counties throughout the U.S. have experienced at least one flooding disaster declaration over the past 30 years and contain a zip code with TRI sites, which makes this a high priority emergency preparedness issue. Further research incorporating disaster loss data, socioeconomic data, and updated flood hazard maps is needed to properly identify areas at high-risk from flood induced TRI releases. Regions with vulnerable populations, flooding hazards, and TRI sites will need additional resources to prevent, prepare, mitigate, respond, and recover from increasingly common extreme weather events.

## Data availability statement

The original contributions presented in the study are included in the article/Supplementary material, further inquiries can be directed to the corresponding author.

## Author contributions

KO: Writing – original draft. SH: Writing – review & editing. AS: Writing – original draft. LQ: Writing – original draft. CL: Writing – review & editing.
